# Electrodeposition Synthesis of Coral-like MnCo Selenide Binder-Free Electrodes for Aqueous Asymmetric Supercapacitors

**DOI:** 10.3390/nano13172452

**Published:** 2023-08-30

**Authors:** Siqi Shao, Song Liu, Changguo Xue

**Affiliations:** 1 School of Materials Science and Engineering, Anhui University of Science and Technology, Huainan 232001, China; suda193@163.com (S.S.); chgxue@foxmail.com (C.X.); 2 Joint National-Local Engineering Research Centre for Safe and Precise Coal Mining, Anhui University of Science and Technology, Huainan 232001, China; 3 CAS Key Laboratory of Mechanical Behavior and Design of Materials, University of Science and Technology of China, Hefei 230026, China

**Keywords:** bimetallic selenide, binder free, coral-like structure, supercapacitors

## Abstract

Bimetallic selenide compounds show great potential as supercapacitor electrode materials in energy storage and conversion applications. In this work, a coral-like MnCo selenide was grown on nickel foam using a facile electrodeposition method to prepare binder-free supercapacitor electrodes. The heating temperature was varied to tune the morphology and crystal phase of these electrodes. Excellent electrochemical performance was achieved due to the unique coral-like, dendritic- dispersed structure and a bimetallic synergistic effect, including high specific capacitance (509 C g^−1^ at 1 A g^−1^) and outstanding cycling stability (94.3% capacity retention after 5000 cycles). Furthermore, an asymmetric supercapacitor assembled with MnCo selenide as the anode and active carbon as the cathode achieved a high specific energy of 46.2 Wh kg^−1^ at 800 W kg^−1^. The work demonstrates that the prepared coral-like MnCo selenide is a highly promising energy storage material.

## 1. Introduction

Due to the ongoing energy crisis and generation of environmental pollution, the investigation of supercapacitors has become a research hotspot. Supercapacitors exhibit many desirable characteristics, such as a rapid charge–discharge rate, high specific power, and excellent cycling stability [1,2,3,4]. To achieve good electrochemical performance, supercapacitors require high-performance electrode materials [5,6]. Transition metal compounds (oxides, sulfides, phosphides, etc.) have been extensively studied as supercapacitor electrode materials, and this field of research is relatively mature [7,8,9,10]. In recent years, transition selenides have attracted widespread attention due to their high electrical conductivity, excellent electrocatalytic activity, and excellent chemical stability [11,12]. Recent theoretical calculations have shown that transition metal Co-based selenide also shows good promise as an energy storage material [13].

Due to the synergistic effects between different metal ions, bimetallic selenides exhibit higher supercapacitor performance compared to monometallic selenides. Numerous studies have introduced other transition metal elements (Ni, Zn, etc.) into cobalt selenide to successfully obtain improved electrochemical performance [14,15,16]. Manganese (Mn) is another transition metal element that can enhance the electrochemical performance of electrode materials due to its multiple oxidation states, low toxicity, and high abundance. Therefore, the introduction of Mn into cobalt selenide shows good promise. The microstructure of electrode materials also significantly impacts their electrochemical performance, and various cobalt-based selenides with novel structures and excellent electrochemical performance have been reported. Chen et al. used high-temperature annealing to prepare a bamboo-like CoSe_2_ array, which provided a high specific capacitance (544.6 F g^−1^ at 1 mA cm^−2^) as a binder-free supercapacitor electrode due to its enhanced electrochemically active surface area [17]. Wang and colleagues used a microwave-assisted technique to prepare a nanoflower-shaped CoSe_2_@NiSe_2_ composite material, which exhibited a high capacitance of 1434 F g^−1^ at 1 A g^−1^ due to its rough surface that provided numerous reaction sites [18]. Sheng et al. synthesized nickel–cobalt selenide compounds with different morphologies using a solvothermal method. Among their prepared samples, those with nanofiber-like structures showed the best electrochemical performance, displaying a high specific capacitance of 1472 mF cm^−2^ at 1 mA cm^−2^ [19]. The existing research on MnCo selenides often involves the preparation of nanosheet structures [20,21], and assembling these nanosheets into dispersed nanostructures is expected to further enhance their electrochemical performance. Traditional electrode fabrication requires the addition of binders, which increases the internal resistance of electrodes. Furthermore, the grinding process used to combine electrode active materials and the binder can damage the electrode morphology. Preparing binder-free electrode materials by directly using conductive substrates (such as nickel foam or carbon cloth) as current collectors offers a potential solution to effectively solve these issues.

In this study, binder-free, coral-like MnCo selenide materials were prepared via a simple and controllable electrodeposition method. The surface morphology and crystal phase structure of the samples were controlled by adjusting the heating temperature. The coral-like structure of these electrode materials exhibits the following advantages: (1) the unique branched and dispersed coral-like morphology facilitates electrolyte infiltration; (2) the main stem of the coral-like structure serves as an electron transmission channel; and (3) the binder-free approach eliminates the use of additives. The optimized MnCo selenide sample achieved a specific capacitance of 509 C g^−1^ at 1 A g^−1^. Moreover, after 5000 cycles at a high current density of 10 A g^−1^, the sample exhibited a capacity retention rate of 94.3%. When assembled with active carbon to form an asymmetric supercapacitor device, a high specific energy of 46.2 Wh kg^−1^ was achieved at a high specific power of 800 W kg^−1^, demonstrating the enormous potential of this coral-like MnCo selenide electrode as an energy storage material.

## 2. Materials and Methods

### 2.1. Synthesis of MnCo Selenide

All chemical reagents were purchased from Sinopharm Chemical Reagent Co., Ltd. (Shanghai, China). The reference and counter electrodes were obtained from Shanghai Chenhua Instrument Co., Ltd. (Shanghai, China). The MnCo selenide samples were prepared with electrodeposition using a three-electrode system. Nickel foam (1 × 1 cm^2^) was used as the working electrode, Ag/AgCl electrode as the reference electrode, and Pt electrode as the counter electrode. Electrodeposition was performed in a clear pink solution consisting of 1.5 mmol MnCl_2_·4H_2_O, 1.5 mmol CoCl_2_·6H_2_O, 6 mmol SeO_2_, and 15 mmol LiCl·H_2_O dissolved in 100 mL of deionized water. The electrochemical deposition process was performed at −0.7 V for 5 min to obtain a MnCo selenide electrode sample, which was dried and denoted MCSe-0. To investigate the effect of the electrode phase on its electrochemical performance, the MCSe-0 sample was annealed under a nitrogen atmosphere at 300 °C, 400 °C, or 500 °C for 2 h to obtain samples denoted MCSe-300, MCSe-400, and MCSe-500, respectively.

### 2.2. Characterization

The surface morphology of the samples was characterized using a scanning electron microscope (SEM, Regulus 8100, Hitachi, Ltd., Tokyo, Japan) at an operating voltage of 20 kV. The microstructure and surface element distribution (EDX spectra, Thermo Scientific, Waltham, MA, USA) of the samples were evaluated using a field-emission transmission electron microscope (STEM, FEI Tecnai G2 F20, Thermo Scientific, Waltham, MA, USA). Crystal phases were identified with X-ray diffractometry (XRD, Smartlab SE, Rigaku Corporation, Tokyo, Japan) over a range from 40). The elemental composition and oxidation states of the prepared samples were evaluated using X-ray photoelectron spectroscopy (XPS, ESCALAB 250Xi, Thermo Scientific, Waltham, MA, USA). Prior to XRD and XPS analysis, powder samples were obtained by ultrasonically stripping the electrode materials from their nickel foam substrates to avoid substrate interference.

### 2.3. Electrochemical Measurements

Cyclic voltammetry (CV), galvanostatic charge-discharge (GCD), and electrochemical impedance spectroscopy (EIS) tests were performed using a CHI 660E electrochemical workstation with a three-electrode system to investigate the electrochemical performance of the individual electrodes. Each prepared, binder-free MnCo selenide electrode served as the working electrode, a Pt electrode was the counter electrode, and a Hg/HgO electrode was the reference electrode, in 6 M KOH electrolyte. The cycling performance of the electrodes was tested using a battery testing system (LAND CT2001A). A two-electrode system in the same instrument was used to evaluate the electrochemical performance of an asymmetric supercapacitor device prepared using the MnCo selenide electrode and active carbon (AC) as the anode and cathode, respectively. The formulas for the mass balance of positive and negative electrodes, as well as the calculation formulas for device energy density and power density, can be found in the supporting information.

## 3. Results

### 3.1. Structure Characterization

**Figure 1:**
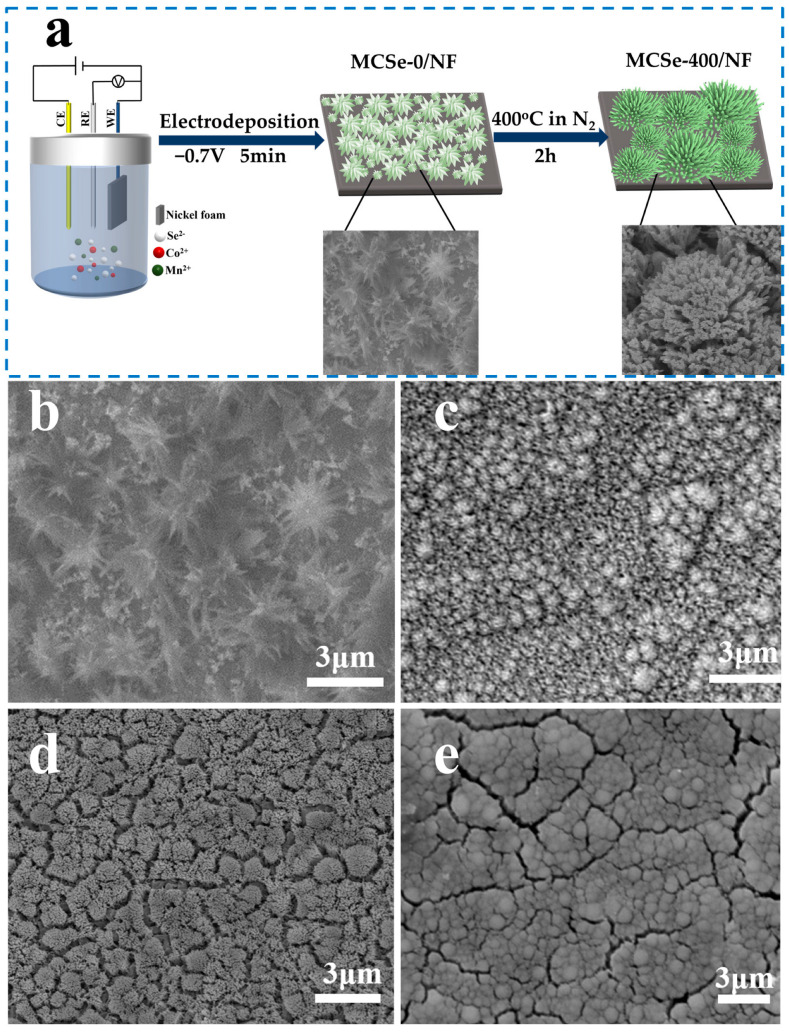
(**a**) Schematic diagram of the manganese cobalt bimetallic selenide electrode preparation process and SEM images of (**b**) MCSe-0, (**c**) MCSe-300, (**d**) MCSe-400, and (**e**) MCSe-500.

**Figure 2:**
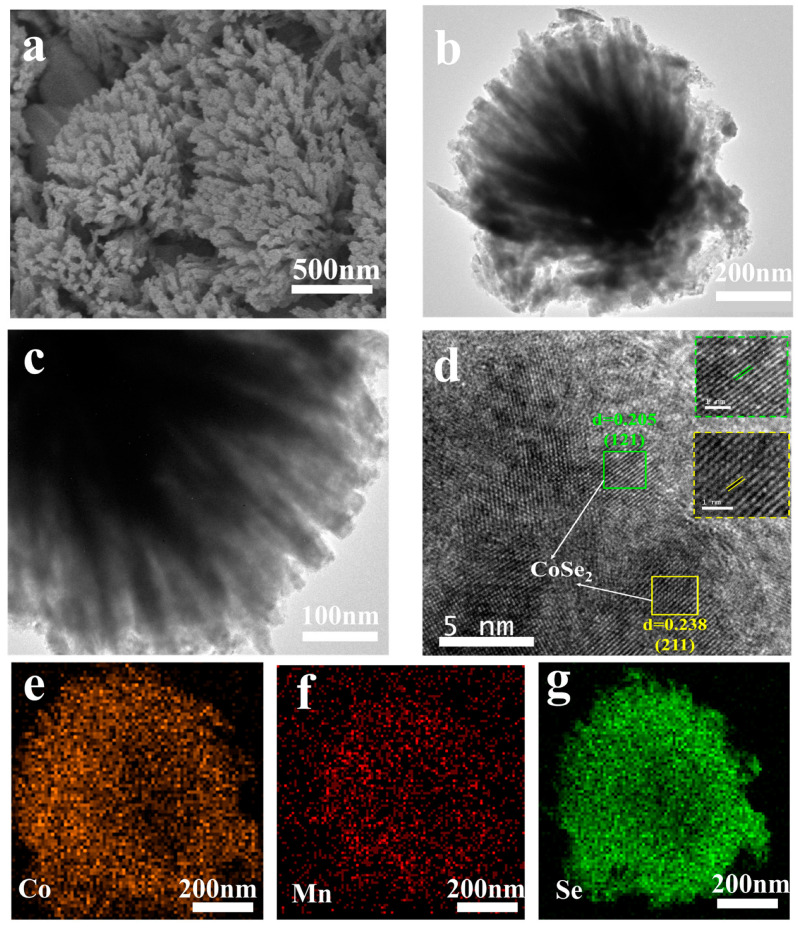
Morphology and elemental analysis of MCSe-400: (**a**) FESEM image, (**b**,**c**) TEM images, (**d**) HRTEM image, and (**e**–**g**) EDS element mapping images.

Figure 1a illustrates the electrodeposition and heating process used to prepare the MnCo bimetallic selenide electrodes. First, when SeO_2_ is dissolved in water, H_2_SeO_3_ is formed via a hydrolysis reaction. Subsequently, H_2_SeO_3_ is reduced to Se_x_^2−^ by constant voltage electrodeposition at −0.7 V. The H_2_SeO_3_ is then reduced to Se_x_^2−^ via a hydrolysis reaction. Then Co^2+^ and Mn^2+^ ions from CoCl_2_·6H_2_O and MnCl_2_·4H_2_O combine with Se_x_^2−^ to form MnCo-Se [22,23]. SEM analysis was used to explore the influence of heating on the surface morphology of the electrode materials, as shown in Figure 1b–e. Initially, MCSe-0 exhibits a coral-like structure composed of nanowire clusters uniformly distributed on the nickel foam substrate. However, high-temperature treatment causes these needle-shaped nanowire structures to partially aggregate into a rod-like morphology. The MCSe-300 sample loses its three-dimensional needle-shaped structure and exhibits an aloe vera-like morphology. When the treatment temperature is increased to 400 °C, the needle-shaped structures shrink and transform into coral-like structures (high-resolution image of the MCSe-400 sample can be seen in Figure 2a). After sintering at 500 °C, the fine structure of the product further aggregates and collapses, resulting in the disappearance of the coral-like structure.

TEM analysis was performed to further study the microstructure of the MCSe-400 sample. As shown in Figure 2b, MCSe-400 exhibits a coral-like cluster structure, which is in good agreement with a high-resolution FESEM image of the same sample (Figure 2a). A magnified view (Figure 2c) shows that the coral-like clusters are composed of nanorods with lengths of approximately 400 nm and widths of approximately 30 nm. These nanorods are further composed of small particles. The coral-like structure of MCSe-400 results in numerous gaps between the nanorods, facilitating the penetration of electrolyte ions. This shows good promise for increasing the contact area between the electrode material and the electrolyte, resulting in better electrode utilization efficiency. HRTEM images (Figure 2d) show lattice fringes at distances of 0.205 nm and 0.238 nm, which correspond to the (121) crystal plane of orthorhombic CoSe_2_ and the (211) crystal plane of cubic CoSe_2_, respectively [24]. Figure 2e–g shows the elemental mapping images of the MCSe-400 sample, demonstrating that the elements Co, Mn, and Se are uniformly distributed on the surface of the electrode material.

**Figure 3:**
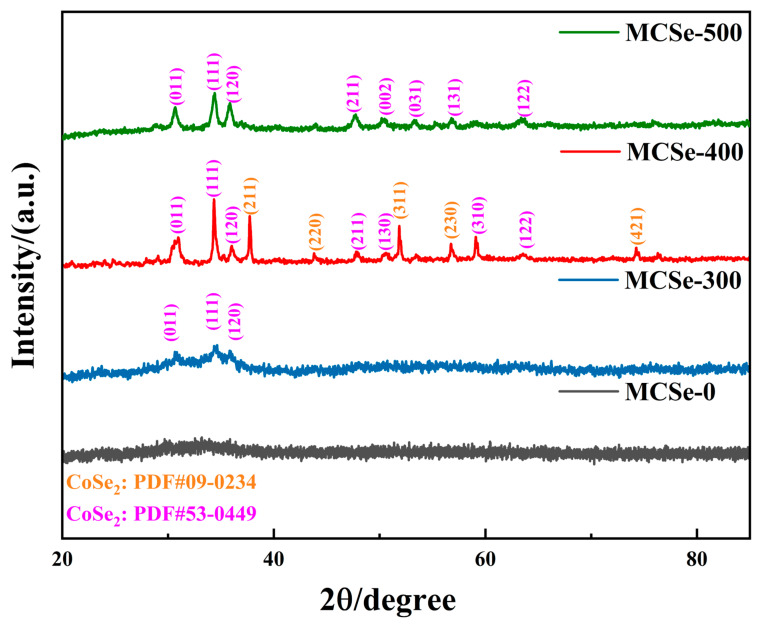
XRD patterns of the MCSe samples.

XRD analysis was used to analyze the crystal structure and composition of the prepared materials. As shown in Figure 3, the MCSe-0 sample does not exhibit any prominent diffraction peaks, indicating its poor crystallinity. However, the samples prepared with high-temperature heating exhibit significantly improved crystallinity. MCSe-300 shows diffraction peaks at 30.7°, 34.5°, and 35.9° corresponding to the (011), (111), and (120) crystal planes of the orthorhombic phase CoSe_2_ (PDF #53-0449), respectively. The MCSe-400 sample, prepared with a higher annealing temperature of 400 °C, shows two distinct sets of diffraction peaks corresponding to two different phases of CoSe_2_: orthorhombic CoSe_2_ (PDF #53-0449) and cubic CoSe_2_ (PDF #09-0234). Among them, the diffraction peaks located at 37.6°, 43.6°, 51.7°, 56.4°, and 73.9° correspond to the (211), (220), (311), (230) and (421) crystal planes of the cubic phase CoSe_2,_ respectively. The diffraction peaks located at 28.9°, 34.5°, 35.9°, 47.7°, 50.6°, 59.2°, and 63.2° correspond to the (011), (111), (120), (211), (130), (310) and (122) crystal planes of orthorhombic phase CoSe_2_, respectively [25]. Moreover, the diffraction peak intensity of this sample is higher than that of MCSe-300, indicating improved crystallinity. After annealing at 500 °C, the MCSe-500 sample only exhibits diffraction peaks ascribed to orthorhombic phase CoSe_2_ (PDF #53-0449) [26].

**Figure 4:**
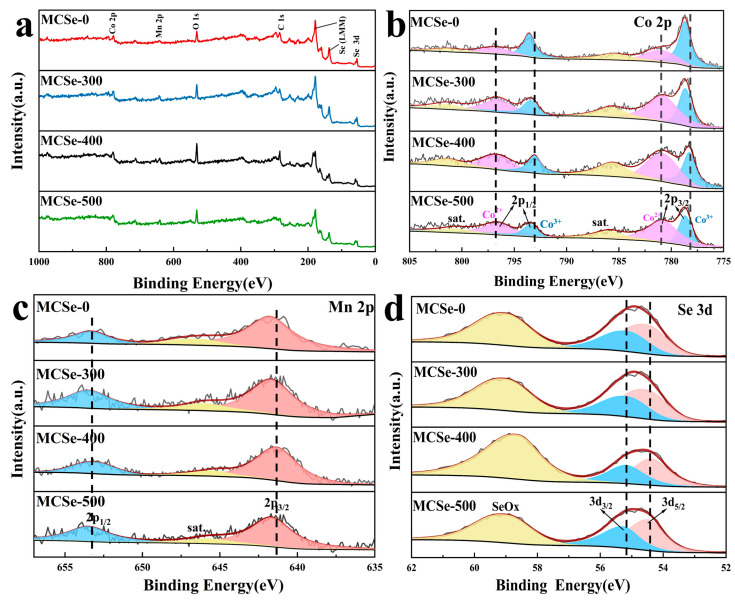
XPS analysis of the MCSe samples: (**a**) survey, (**b**) Co 2p, (**c**) Mn 2p, and (**d**) Se 3d spectra.

The prepared electrode materials were evaluated with XPS to investigate their chemical composition and oxidation states, as shown in Figure 4a–d. The survey spectra of these samples display the outer electron orbital peaks and Auger peaks of Co, Mn, and Se (Figure 4a), confirming the presence of these elements. The Co 2p orbital spectra (Figure 4b) show two spin peaks (2p_3/2_, 2p_1/2_) and a pair of satellite vibrational peaks, indicating the presence of Co ions in two different chemical states. In the MCSe-400 spectrum, the peaks located at 778.3 eV (2p_3/2_) and 793.1 eV (2p_1/2_) are attributed to Co^3+^ ions, while the peaks at 781.6 eV (2p_3/2_) and 796.9 eV (2p_1/2_) are assigned to Co^2+^ ions. Additionally, satellite vibration peaks can be observed at 785.1 eV and 801.5 eV [27,28]. Compared to the other MCSe samples, the MCSe-400 sample exhibits the smallest Co^3+^ fitting peak area and the largest Co^2+^ fitting peak area. Therefore, heating at 400 °C increases the content of Co^2+^ ions and enhances the redox capability of the electrode material [29]. The Mn 2p orbital spectrum of MCSe-400 (Figure 4c) shows peaks located at 641.4 eV and 653.2 eV attributed to the Mn 2p_3/2_ and Mn 2p_1/2_ orbitals, respectively. A satellite vibration peak can also be observed at 645.6 eV [30,31], indicating the 2^+^ oxidation state of Mn in this sample. Combined with the XRD results, this indicates the successful incorporation of Mn into the CoSe_2_ lattice. The Se 3d spectrum of MCSe-400 (Figure 4d) shows peaks located at 54.4 eV and 55.2 eV that are attributed to the Se 3d_5/2_ and Se 3d_3/2_ orbitals, respectively. In addition, these peaks are characteristic of metal–selenium bonds. The peak at 58.5 eV is assigned to SeO_x_, whose presence is possibly due to surface oxidation in ambient air [32,33]. The other samples (MCSe-0, MCSe-300, and MCSe-500) have spectra with similar peaks. However, compared to these samples, the orbital peaks in the XPS spectra of MCSe-400 are located at lower binding energies. This can potentially be attributed to the formation of two different CoSe_2_ crystal phases after annealing, resulting in changes in the lattice parameters that affect the electron density and state of each ion [21].

### 3.2. Electrochemical Performance

**Figure 5:**
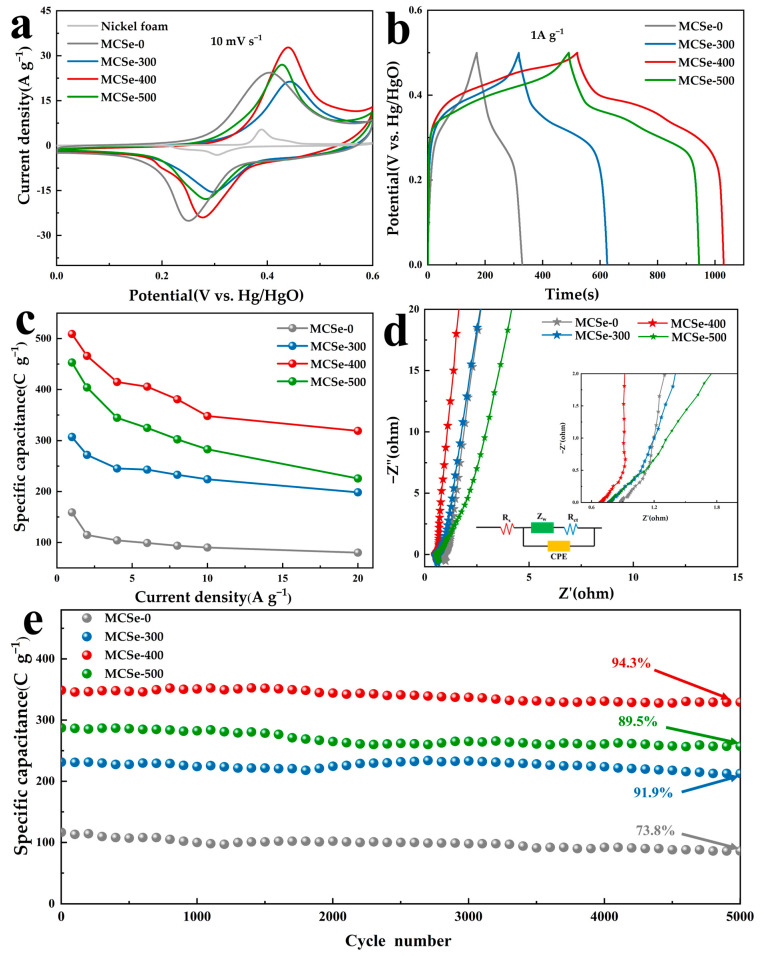
Electrochemical properties of MCSe electrodes: (**a**) CV curves, (**b**) GCD curves, (**c**) specific capacitance, (**d**) EIS curves (inset: EIS circuit diagram), and (**e**) long-term stability across 5000 cycles.

Figure 5a shows the CV curves of all the MCSe electrodes, and a pair of redox peaks can be observed for each electrode. The MCSe-400 electrode has a larger CV integral area, indicating higher specific capacitance compared to the other electrodes. A CV curve of the bare nickel foam substrate was also obtained. The integration area region of this CV curve is much smaller than the integration area of the CV curves of the MCSe electrodes, which indicates that the nickel foam substrate has a negligible effect on the electrochemical performance. GCD tests were conducted at 1 A g^−1^, as shown in Figure 5b. MCSe-400 demonstrates a longer discharge time than the other electrode samples, further confirming its superior electrochemical performance. The specific discharge capacities of all MCSe electrodes were calculated from the GCD curves as a function of current density, as shown in Figure 5c. At a current density of 1 A g^−1^, the discharge-specific capacity of the MCSe-400 electrode reaches 509 C g^−1^. When the current density was increased to 10 A g^−1^, the capacitance was retained by 68.6% (higher than the other MCSe electrodes), indicating the good rate capability of the MCSe-400 electrode. The specific capacitance values of the MCSe electrodes calculated on the basis of electrode area can be seen in Figure S1, where the maximum area specific capacitance of 1572 mF cm^−2^ is obtained for MCSe-400 at a current density of 1.5 mA cm^−2^. EIS tests were also performed, and the obtained Nyquist plots are shown in Figure 5d. As shown in the EIS circuit model, the intersection of the high-frequency region with the X-axis represents the electrolyte solution resistance (R_s_), and the MCSe-400 electrode has the lowest internal resistance. The small, indistinct semicircle in these curves corresponds to the charge transfer resistance (R_ct_). The MCSe-400 electrode exhibits an R_ct_ value of 0.53 Ω, which is lower than that of MCSe-0 (0.76 Ω), MCSe-300 (0.89 Ω), and MCSe-500 (0.98 Ω). This demonstrates the good conductivity of MCSe-400 compared to the other electrodes, which facilitates a rapid charge transfer. Compared to the other electrodes, the MCSe-400 electrode exhibits the steepest slope in the low-frequency region, indicating the lowest Warburg impedance (Z_w_), which in turn suggests that the electrode has the highest ion diffusion rate. To evaluate their long-term stability, the electrodes were tested for 5000 testing at 10 A g^−1^ (Figure 5e). After 5000 cycles, the MCSe-400 electrode retained 94.3% of its initial capacity, which was higher than that of the MCSe-0 (73.8%), MCSe-300 (91.9%), and MCSe-500 (89.5%) electrodes. The SEM image of the MCSe-400 electrode after long cycling can be seen in Figure S2. The coral-like structure can be noticed collapsed, which may be the reason for the capacity decay. In summary, the MCSe-400 electrode exhibits better stability than the other electrode materials. The optimal electrochemical performance of the MCSe-400 electrode material may be attributed to the fact that it contains orthorhombic phase CoSe_2_ which has more active sites and supports stronger OH^−^ ion adsorption, thus providing a high energy storage capacity, and that it is a polycrystalline CoSe_2_ containing two crystalline phases, which has higher catalytic activity and better electrochemical performance than the pure orthorhombic phase CoSe_2_. The above are consistent with the results of existing theoretical calculations and experimental conclusions [23,25].

**Figure 6:**
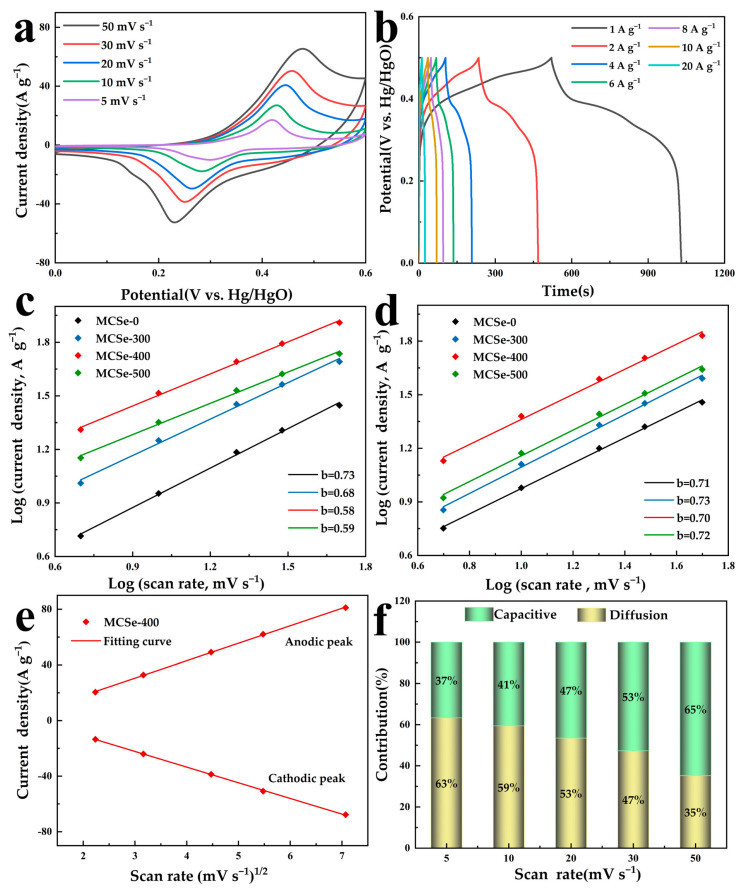
Electrochemical performance of the MCSe-400 electrode: (**a**) CV curves; (**b**) GCD curves; (**c**) fitted plots of anode peak response current log(i_p_) versus log(v) of MCSe electrodes; (**d**) fitted plots of cathode peak response current log(i_p_) versus log(v) of MCSe electrodes; (**e**) fitted curves of peak current versus the square root of the scan rate of MCSe-400 electrode; and (**f**) volume contribution ratios of MCSe-400 electrode at different scan rates.

CV curves were also obtained at different scan rates, as shown in Figure 6a. With an increasing scan rate, the ion diffusion rate decreases, meaning that fewer ions are available for the interfacial reaction. Consequently, the oxidation and reduction current peak positions shift toward both ends of the scanning direction. Figure 6b displays the GCD curves of the MCSe-400 electrode under different current densities. All the GCD curves exhibit good symmetry, indicating the high electrochemical reversibility of MCSe-400. The charge storage mechanism of the electrodes is attributed to two components, the diffusion-controlled capacitance and surface-controlled capacitance, which can be determined using the following function:

(1) $$i=av^{b}$$

where *i* represents the peak current, v is the scan rate, and *a* and *b* are the constants. A value of *b* = 0.5 indicates diffusion-controlled behavior, while a value of *b* = 1 indicates that charge storage occurs via surface-controlled processes. The anodic and cathodic curves of MCSe electrodes were fitted, and the *b* values were obtained, as shown in Figure 6c,d. This analysis suggests that the pseudocapacitive behavior of the MCSe electrode materials involves both diffusion-controlled and surface-controlled processes. In addition, the percentage contribution of surface capacitance-controlled and diffusion-controlled processes at a given scan rate is distinguished according to the following equation:

(2) $$i_{p}=k_{1}v+k_{2}v^{1∕2}.$$

Figure 6e shows the fitted curve of peak current versus square root of scan rate of the MCSe-400 electrode. The adjustable parameters *k*_1_ and *k*_2_ in the surface capacitance current ($k_{1}v$) and diffusion control current ($k_{2}v^{1∕2}$) can be obtained from the curves. A calculation of the ratio between diffusion-controlled current and total current is shown in Figure 6f. Based on Figure 6f, the pseudocapacitive behavior of MCSe-400 is predominantly diffusion-controlled [34,35].

### 3.3. Electrochemical Performance of MCSe-400//AC Asymmetric Supercapacitor

**Figure 7:**
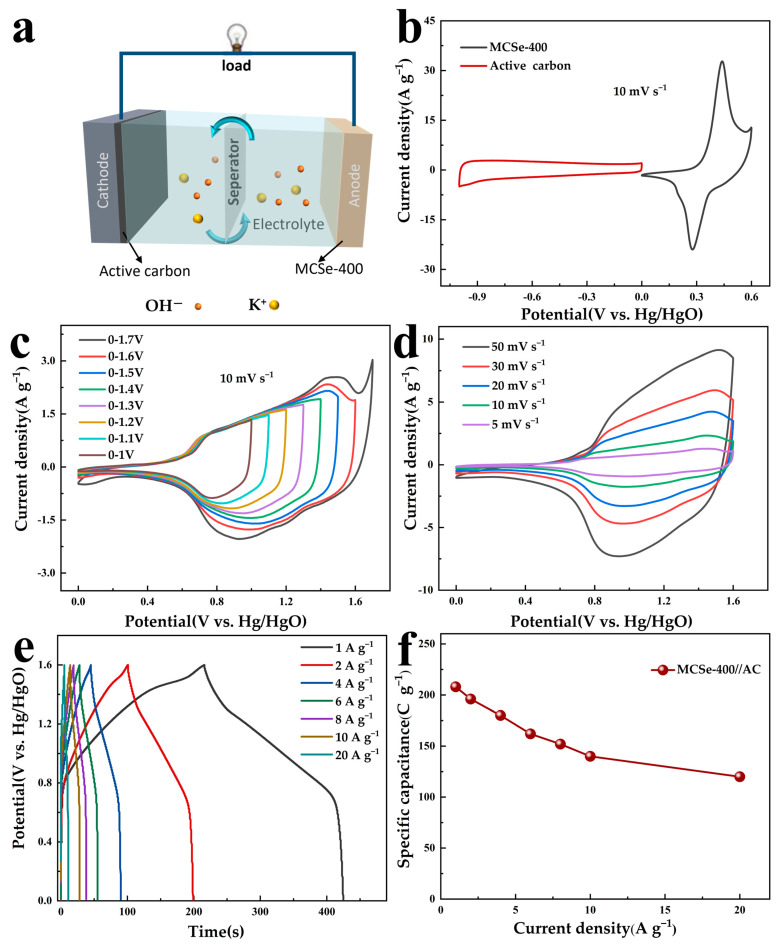
(**a**) Schematic diagram of MCSe-400//AC device structure. Electrochemical performance of MCSe-400//AC ASC: (**b**) CV curves of AC and MCSe-400 electrodes obtained at a scan rate of 10 mV s^−1^, (**c**) CV curves o at different voltage intervals with a scan rate of 10 mV s^−1^, (**d**) CV curves, (**e**) GCD curves, and (**f**) specific capacity at different current densities.

To further evaluate the practical application of the prepared MCSe-400 material, an asymmetric supercapacitor (ASC) device was assembled using MCSe-400 as the anode and active carbon (AC) as the cathode. A schematic diagram of the ASC structure is shown in Figure 7a. The CV curves of the AC (−1 to 0 V) and MCSe-400 (0 to 0.6 V) electrodes in a three-electrode system at a scan rate of 10 mV s^−1^ are displayed in Figure 7b. The AC electrode exhibits rectangular-like behavior, while the MCSe-400 electrode shows distinct redox peaks. Therefore, the positive and negative electrodes of the assembled ASC have different charge storage mechanisms.

**Figure 8:**
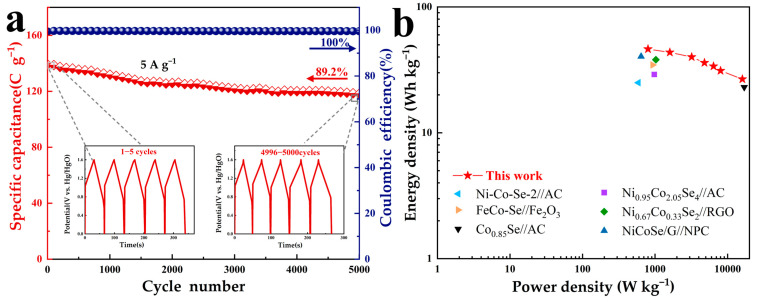
(**a**) Cyclic stability and Coulombic efficiency ASC after 5000 charge/discharge cycles at 5 A g^−1^ (inset: the charge/discharge curves of the first five cycles and the last five cycles) and (**b**) Ragone plot.

To achieve excellent charge storage performance, a charge balance (Q^+^ = Q^−^) was performed to determine that the optimized mass ratio of MCSe-400 to AC is 2.01. The charge balance formula is found in the supporting information. As shown in Figure 7c, under a scan rate of 10 mV s^−1^, CV curves with similar shapes are obtained and no significant polarization is observed when the potential window is expanded from 1 V to 1.6 V. However, when the potential window is further expanded to 1.7 V, noticeable polarization can be observed. Therefore, the voltage range of this ASC device was selected to be 0–1.6 V. Figure 7d shows the CV curves of the MCSe-400//AC device at different scan rates. Well-shaped CV curves are obtained under scan rates of 5–50 mV s^−1^, indicating that the MCSe-400//AC ASC device exhibits good rate performance. GCD curves of the MCSe-400//AC device were obtained at current densities ranging from 1 to 20 A g^−1^, as shown in Figure 7e. Charge–discharge plateaus are visible in all the GDC curves, which is consistent with the formation mechanism of the oxidation and reduction peaks in the CV curves. The specific capacitance of the MCSe-400//AC device is 208 C g^−1^ at 1 A g^−1^. Even at a high current density of 20 A g^−1^, a specific capacitance of 120 C g^−1^ can still be maintained (Figure 7f), indicating the excellent rate performance of this device. The cycling stability of the MCSe-400//AC ASC was evaluated over 5000 cycles at 5 A g^−1^, as shown in Figure 8a. After testing, the MCSe-400//AC ASC still retains 89.2% of its initial specific capacitance, indicating good cycling stability. Throughout this cycling test, the Coulombic efficiency of the electrode remains close to 100%, indicating the good reversibility of the surface redox reactions. The charge–discharge curves for the cycles of 1–5 and 4996–5000 are shown in the Figure 8a inset. The charge–discharge region of this curve shows clear bending, indicating that the capacity of this electrode originates from the redox reactions. No significant changes can be observed in the curve shape even after 5000 cycles, further confirming the excellent cycling performance of the MCSe-400//AC ASC. The specific energy and specific power of the MCSe-400//AC device were also calculated. As shown in the Ragone plot (Figure 8b), the MCSe-400//AC device exhibits specific energy of 46.2, 43.5, 36, 31.1, and 26.6 Wh kg^−1^ at specific powers of 800, 1600, 5000, 8000, and 16,000 W kg^−1^, respectively. The specific energy and specific power calculation formulas can be found in the supporting information. Overall, the performance of the MCSe-400//AC device surpasses that of other dual-transition metal selenide supercapacitor devices [36,37,38,39,40,41].

## 4. Conclusions

Coral-like MnCo selenide electrode materials were directly grown on nickel foam by electrodeposition. The morphology and crystal structure of the prepared MnCo selenide electrodes were optimized by changing the heating temperature. Performing electrodeposition at −0.7 V and 5 min followed by heating at 400 °C (MCSe-400) led to better conductivity and a highest specific capacitance (509 C g^−1^ at 1 A g^−1^). Moreover, the MCSe-400 electrode exhibited an excellent cycling performance (94.3% after 5000 cycles). Its polycrystalline CoSe_2_ improves the ion adsorption capacity and catalytic activity and, therefore, obtains the optimal electrochemical performance. An asymmetric supercapacitor device prepared with MCSe-400 and AC as two electrodes also showed good electrochemical performance, with a high specific energy of 46.2 Wh kg^−1^ even at a high power of 800 W kg^−1^, thus demonstrating the proof that MCSe-400 is a promising electrode material for supercapacitors.

## Data Availability

Data will be available on request.

## Supplementary Materials

The following supporting information can be downloaded at: https://www.mdpi.com/article/10.3390/nano13172452/s1, Figure S1: Specific capacitance based on MCSe electrode area; Figure S2: SEM images of MCSe-400 after 5000 cycles: (a) low magnification image, (b) high magnification image.
