# Supplementary material for: Electrodeposition Synthesis of Coral-like MnCo Selenide Binder-Free Electrodes for Aqueous Asymmetric Supercapacitors
Source: Nanomaterials (Basel). 2023 Aug 30;13(17):2452. doi: 10.3390/nano13172452 (PMC10489885; doi:10.3390/nano13172452)
Supplement: Supplementary file 1 [file nanomaterials-13-02452-s001.zip › nanomaterials-2586683-supplementary.pdf]

Supporting Information

1 → The mass ratio of MCSe-400 to AC is determined according to the following formula:

$$m^{+}/m^{-} = (C_s^{-} * \Delta V^{-})/(C_s^{+} * \Delta V^{+}) \quad (1)$$

Where m^+, C_s^+ and ΔV^+ are the mass (g), specific capacitance (C g^{-1}) and corresponding voltage interval (V) of the MCSe-400 electrode, respectively; m^-, C_s^- and ΔV^- are the mass (g), specific capacitance (C g^{-1}) and corresponding voltage interval (V) of AC electrode, respectively.

2 → The specific energy (E, Wh Kg^{-1}) and specific power (P, W Kg^{-1}) of the device were calculated basing on the equations below:

$$E = \frac{C_S \Delta V^2}{7200} \quad (2)$$

$$P = \frac{3600E}{t} \quad (3)$$

The C_s of the ASC was the achieved from the same equation of electrode except that the m contains the total mass of cathode and anode active materials.

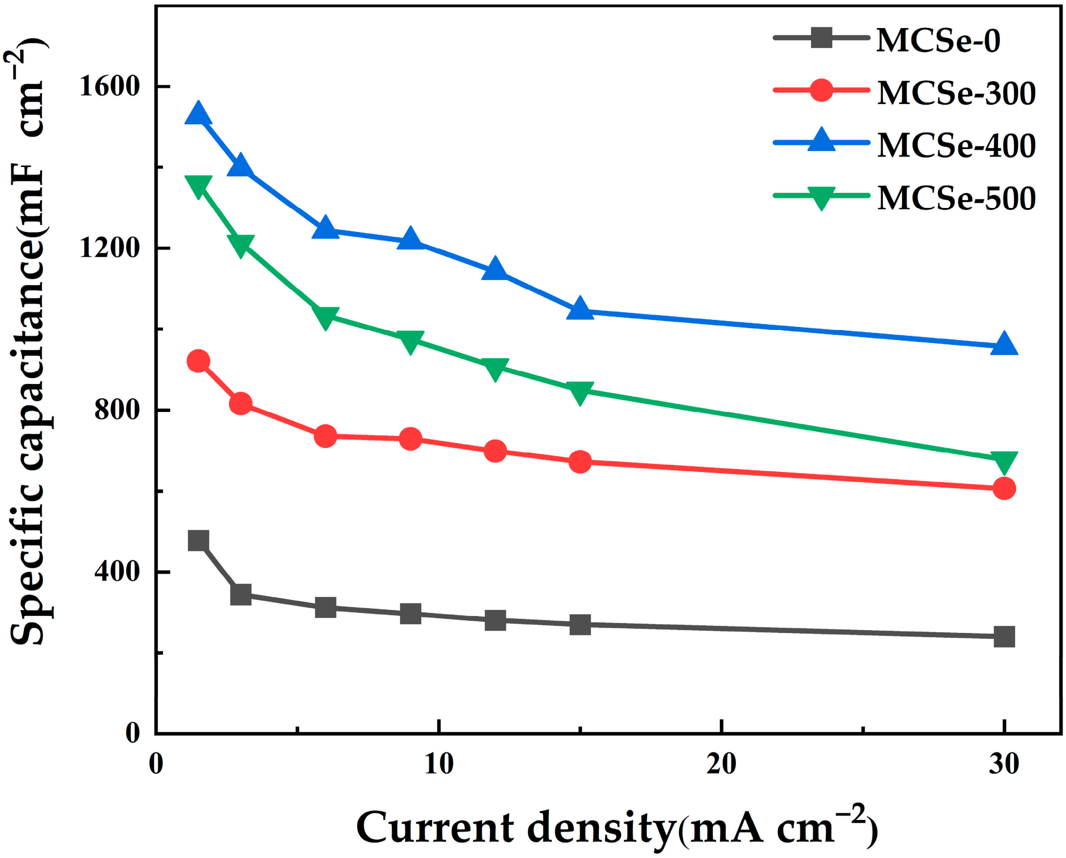

Figure S1. Specific capacitance based on MCSe electrode area

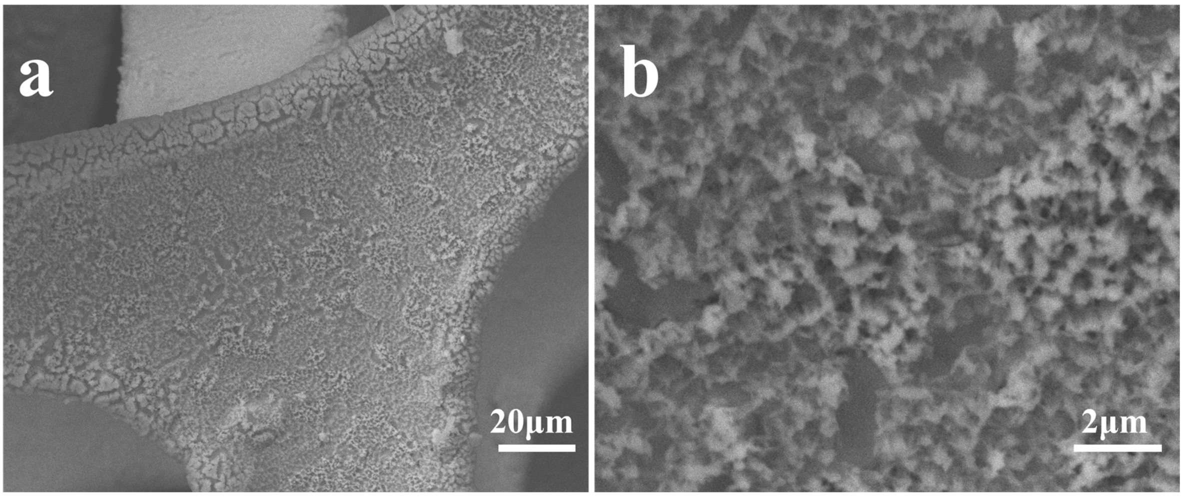

Figure S2. SEM images of MCSe-400 after 5000 cycles: (a) low magnification image, (b) high magnification image.
